# Mortality Burden and Socioeconomic Status in India

**DOI:** 10.1371/journal.pone.0016844

**Published:** 2011-02-09

**Authors:** June Y. T. Po, S. V. Subramanian

**Affiliations:** 1 Department of Global Health and Population, Harvard School of Public Health, Boston, Massachusetts, United States of America; 2 Department of Society, Human Development and Health, Harvard School of Public Health, Boston, Massachusetts, United States of America; Erasmus University Rotterdam, Netherlands

## Abstract

**Background:**

The dimensions along which mortality is patterned in India remains unclear. We examined the specific contribution of social castes, household income, assets, and monthly per capita consumption to mortality differentials in India.

**Methods and Findings:**

Cross-sectional data on 217 363 individuals from 41 554 households from the 2004–2005 India Human Development Survey was analyzed using multiple logistic regressions. Mortality differentials across social castes were attenuated after adjusting for household economic factors such as income and assets. Individuals living in the lowest income and assets quintiles had an increased risk of mortality with odds ratio (OR) of 1.66 (95% CI  = 1.23–2.24) in the bottom income quintile and OR of 2.94 (95% CI  = 1.66–5.22) in the bottom asset quintile. Counter-intuitively, individuals living in households with lowest monthly consumption per capita had significantly lower probability of death (OR  = 0.27, 95% CI  = 0.20–0.38).

**Conclusions:**

Mortality burden in India is largely patterned on economic dimensions as opposed to caste dimensions, though caste may play an important role in predicting economic opportunities.

## Introduction

Social class and economic well-being have been identified as important social determinants that shape health inequalities [1], [2], [3], [4], [5]. In India, social castes were previously considered as a proxy for socioeconomic status and poverty [6]. A nationally representative study on India based on the 1981 census indicated that under-five child mortality in the lower castes, Scheduled Tribes and Scheduled Castes, were significantly higher than upper social castes [7]. The 1998–1999 India National Family Health Survey (NFHS) demonstrated an increase in mortality rates of neonates, infants, and under five children in progressively disadvantaged social castes [8], [9]. In more detail, a study on an urban population of North India provided evidence that cardiovascular-related mortality was more prevalent in higher social castes whereas infections-related mortality was more prevalent in lower social castes [10]. Similarly, based on the analysis of the 1998-99 NFHS data, mortality was found to differentially associate with household wealth and much less with caste [8]. In this study, we provide an update of the most recent association between social caste, economic factors and mortality in India using individual data from the India Human Development Survey 2004–2005.

## Methods

### Study Design and Data

The cross-sectional data was drawn from the India Human Development Survey (IHDS) [11], a nationally representative, multi-topic survey collected from November 2004 to October 2005. It sampled 215 754 alive individuals from 41 554 rural and urban households in India. Villages and urban blocks formed the primary sampling unit consisted of 150–200 households, from which the sample of households was selected [11]. The survey response rates were calculated as 92% for the total sample [11].

The lowest unit of observation was the individual member, including 1609 who died in the previous year. Data on age and gender for both living and deceased household members were collected. The deceased household members were assumed to have belonged in the similar caste and religion as other household members and benefited from similar quality of living based on shared household income, assets and consumption. One household was defined as a group of people living under one roof and sharing the same kitchen.

### Outcome and Predictors

The study outcome measure was a dichotomous variable indicating whether an individual was dead (1) or alive (0).

Caste and religion of the household were self-identified by the head of household. The caste categories were separated into Brahmin, High Caste, Other Backward Classes, Scheduled Castes (Dalit), Scheduled Tribes (Adivasi), and No Caste. Other Backward Classes, Dalits and Adivasis are considered the lower, marginalized social groups in India [12]. The individuals in IHDS that self-identified as No Caste were further stratified according to their religion into Muslims, Christians and Sikhs and Jains combined.

Detailed household income data was collected from queries of over 50 different income sources. The queries were categorized into eight major household income types: family farm income, household agricultural wages, non-agricultural wages, salaries, net business income, sum household remittance, government benefits, and property and pensions. The current analysis used the aggregated total income data and divided it into quintile groups and a group that reported negative household income.

The variable for household assets was a score constructed from the summation of 22 equally weighted dichotomous items measuring household possessions of consumer goods and eight aspects of housing quality. The household asset score was divided into quintiles for the current analysis.

The consumption variable was constructed from a standard battery of 47 expenditure questions taken from the short form of India's National Sample Survey. These included 30 questions on monthly expenditure and 17 questions on annual expenditure reported for the previous year. The final consumption total was calculated as the sum of the expenditure on monthly items and one twelfth of the expenditure on annual items. The monthly consumption per capita was divided into quintiles for the current analysis.

Age was divided into six categories to capture the different stages of life course: infants (<1 year), young children (1–5 years), children or adolescents (6–18 years), young adults (19–44 years), middle-aged adults (45–64 years) and elderly ( = 65 years). Other predictors were gender and residency location. Residency location was divided into three categories: rural villages, urban neighborhoods that were not metropolitan cities (population 5000–100 000) and cities of Mumbai, Delhi, Kolkata, Chennai, Bangalore and Hyderabad (population >100 000). The data on residency location was based on the India Census 2001, where an urban neighborhood must have a minimum population of 5000, at least 75% of male working population engaged in non-agricultural pursuits and a population density of at least 400 persons per square kilometer.

### Statistical Analysis

We used logistic regression to model the association between mortality at the individual level with demographic and socioeconomic predictors. The binary response (y, dead or not) for each individual were related to a set of categorical predictors, X, (gender, age, residency location, religion, caste, income, assets, monthly consumption per capita) and a fixed state effect by a logit link function:
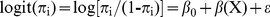
(1)


The probability of an individual being dead is π_i_. The parameter β_0_ estimates the log odds of mortality for the reference group, and the parameter β estimates with maximum likelihood, the differential log odds of mortality associated with the predictor X, as compared to the reference group. Odds ratios (OR) and predicted probabilities (PP) with 95% CI were calculated. All analyses were performed using the statistical program SAS 9.2 ‘surveylogit’ procedure, adjusted for sample clustering at the level of primary sampling units.

We used multivariable regression models to explore the effects of social caste, household income, household assets, and monthly consumption per capita on mortality separately, while adjusting for gender, age and residency location. Secondly, we explored the associations of the above factors together as they mutually adjusted for each other. Furthermore, we explored these associations with regards to age-specific mortality by adding interaction terms between age and caste, income, assets and monthly consumption per capita. We also explored the effects of caste on mortality when modified by income, assets and monthly consumption per capita. Social castes were stratified into five groups to observe mortality differentials across asset quartiles in finer detail. Households were re-grouped into asset quartiles to ensure at least one death is present within each asset quartile stratified by caste.

### Ethical Review

The India Human Development Survey was conducted under the scientific and administrative supervision of the National Council of Applied Economic Research, Delhi and the University of Maryland and was reviewed by the relevant ethics review board. Formal written consent was obtained for all the surveys. This study was reviewed by Harvard School of Public Health Institutional Review Board and was considered exempt from full review as it was based on an anonymous public use data set with no identifiable information on survey participants.

## Results

### Descriptive Statistics

There were 217 363 individuals included in the analysis. There were 1609 individuals, 0.7% of the total sample, who died in the one year prior to household survey. The distributions of gender, religion, social castes, and quintiles of household income, household assets and monthly consumption per capita are listed in Table 1.

**Table 1:** Number and Percentage of Deaths during One Year Before the Survey, by Descriptive Characteristics of the Sample: India Human Development Survey, 2004-2005.

	N	(%)	Deaths	(%)	(95% CI)
**Total**	217363	100	1609		
**Gender**					
Men	110765	50.96	960	1.00	(0.84–1.15)
Women	106598	49.04	649	0.74	(0.65–0.83)
**Age**					
Infants (<1 y)	3184	1.46	100	3.61	(2.62–4.61)
Young children (1–5 y)	21332	9.81	102	0.60	(0.41–0.78)
Children/Adolescents (6–18 y)	63523	29.22	88	0.17	(0.12–0.21)
Young adults (19-44 y)	83167	38.26	236	0.33	(0.26–0.39)
Middle-aged adults (45–64 y)	33817	15.56	352	1.21	(0.98–1.44)
Elderly ( = 65 y)	12340	5.68	731	6.99	(5.80–8.18)
**Caste**					
Brahmin	12207	5.62	99	1.07	(0.61–1.52)
High caste	35748	16.45	217	0.66	(0.48–0.84)
Other Backward Classes	73481	33.81	581	0.90	(0.80–1.01)
Scheduled Castes (Dalit)	43618	20.07	348	1.08	(0.77–1.40)
Scheduled Tribes (Adivasi)	17541	8.07	136	0.80	(0.61–1.00)
No caste	34768	16.00	228	0.67	(0.56–0.79)
**Religion**					
Hindu	165054	75.93	1245	0.92	(0.80–1.03)
Muslim	27841	12.81	180	0.67	(0.54–0.80)
Sikh, Jain	3691	1.70	19	0.44	(0.23–0.65)
Christian	3236	1.49	29	0.88	(0.49–1.26)
Other religion	17541	8.07	136	0.80	(0.61–1.00)
**Urban-Rural Status**					
Metro city	19329	8.89	72	0.74	(0.16–1.31)
Small city or town	57687	26.54	420	0.81	(0.63–1.00)
Village	140347	64.57	1117	0.91	(0.82–1.00)
**Income**					
Top quintile	57585	26.49	327	0.64	(0.53–0.75)
Second quintile	48157	22.16	293	0.58	(0.47–0.70)
Third quintile	40720	18.73	291	0.87	(0.71–1.03)
Fourth quintile	35494	16.33	302	1.14	(0.78–1.49)
Bottom quintile	31412	14.45	351	1.23	(1.05–1.41)
Negative income	3995	1.84	45	1.12	(0.67–1.58)
**Household Assets**					
Top quintile	5456	2.51	33	0.65	(0.39–0.90)
Second quintile	36422	16.76	198	0.54	(0.44–0.63)
Third quintile	58707	27.01	368	0.69	(0.55–0.82)
Fourth quintile	72084	33.16	589	1.03	(0.83–1.23)
Bottom quintile	44694	20.56	421	1.01	(0.87–1.15)
**Monthly Consumption per Capita**					
Top quintile	43516	20.02	446	1.20	(0.96–1.45)
Second quintile	43411	19.97	324	0.89	(0.72–1.07)
Third quintile	43602	20.06	295	0.72	(0.61–0.83)
Fourth quintile	43546	20.03	304	1.02	(0.72–1.31)
Bottom quintile	43288	19.92	240	0.60	(0.48–0.73)

### Socioeconomic Differentials in Mortality

The conditional odds ratios (OR) and predicted probabilities (PP) of each subgroup is shown in Table 2 and Table S1. The reference group represents a Hindu male between the ages of 19–44, living in a metropolitan city. He belongs to the High Caste and has household income, household assets and monthly consumption per capita of the highest quintile.

**Table 2:** Odds Ratios of Mortality by Socioeconomic Factors, Adjusted for Gender, Age, Urban-Rural Status, Religion, Fixed Effects on States: Indian Human Development Survey, 2004-2005.

	Unadjusted for SES factors		Adjusted for SES factors	
	OR	(95% CI)	OR	(95% CI)
**Caste**				
Brahmin	1.50	(0.89–2.53)	1.54	(0.92–2.57)
High caste	1.00		1.00	
Other Backward Classes	1.47	(1.09–1.98)*	1.33	(0.99–1.79)
Scheduled Castes	1.99	(1.36–2.92)*	1.72	(1.23–2.41)*
Scheduled Tribes	1.47	(1.02–2.13)*	1.37	(0.95–1.98)
No caste (Muslim)	1.30	(0.91–1.87)	1.16	(0.81–1.66)
No caste (Sikh, Jain)	0.63	(0.36–1.09)	0.75	(0.43–1.31)
No caste (Christian)	1.06	(0.61–1.82)	1.16	(0.70–1.91)
**Income**				
Top quintile	1.00		1.00	
Second quintile	0.93	(0.70–1.22)	0.92	(0.68–1.23)
Third quintile	1.43	(1.09–1.86)*	1.36	(1.01–1.83)*
Fourth quintile	1.90	(1.25–2.87)*	1.81	(1.20–2.72)*
Bottom quintile	1.76	(1.35–2.29)*	1.66	(1.23–2.24)*
**Household Assets**				
Top quintile	1.00		1.00	
Second quintile	0.96	(0.62–1.51)	1.06	(0.67–1.67)
Third quintile	1.39	(0.85–2.27)	1.74	(1.02–2.98)*
Fourth quintile	2.32	(1.32–4.08)*	2.93	(1.65–5.22)*
Bottom quintile	2.38	(1.42–3.99)*	2.94	(1.66–5.22)*
**Monthly Consumption per Capita**				
Top quintile	1.00		1.00	
Second quintile	0.78	(0.59–1.02)	0.60	(0.44–0.81)*
Third quintile	0.62	(0.48–0.81)*	0.39	(0.30–0.53)*
Fourth quintile	0.91	(0.59–1.38)	0.49	(0.34–0.71)*
Bottom quintile	0.54	(0.40–0.74)*	0.27	(0.20–0.38)*

*significance with *p*-value <0.05.

Across social castes, we found significantly higher odds of mortality in Other Backward Classes, Scheduled Castes and Scheduled Tribes. However, after adjusting for all wealth factors: income, assets and consumption per capita, the associations was no longer statistically significant with the exception of Scheduled Castes (OR  = 1.72, 95% CI  = 1.23–2.41). Adjusting the effect of social caste on mortality with household income and asset ownership independently also resulted in the attenuation of caste effect on mortality except in Scheduled Castes (Table S2). Within the Muslims, Christians, Sikhs and Jains populations outside the traditional social caste system, no significant patterning in risk of mortality was found.

We found a statistically significant association between household income and mortality. Compared to the top quintile, the third and fourth quintile displayed significant and progressively higher odds of mortality. Individuals living with household income at the bottom quintile had 76% higher odds of mortality (OR  = 1.76, 95% CI  = 1.35–2.29). Mortality differentials in lower income quintiles were attenuated after mutually adjusting for assets and expenditure, but remained statistically significant (OR  = 1.66, 95% CI  = 1.23–2.24).

Similar to associations with household income, individuals with household assets within the middle quintile and lower quintiles had higher odds of mortality. Individuals who had little or no ownership of household assets at the bottom quintile had odds of mortality substantially higher than the top quintile (OR  = 2.38, 95% CI  = 1.42–3.99), which increased to almost three times when adjusted for income and expenditure (OR  = 2.94, 95% CI  = 1.66–5.22).

Unlike household income and assets ownership, a decrease in monthly consumption per capita was associated with decreased odds of mortality. Individuals living in households with the lowest quintile of monthly consumption per capita were 73% less likely to die than individuals from the top consumption quintile (OR  = 0.27, 95% CI  = 0.20–0.38).

### Interaction of Socioeconomic Factors with Age

**Figure 1:**
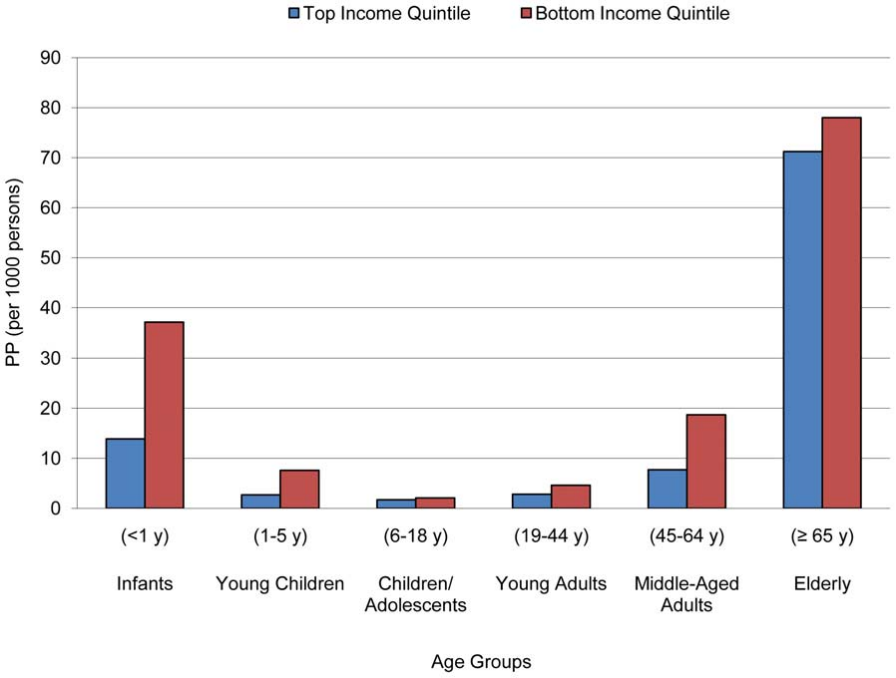
Predicted probabilities (PP) of death by age groups comparing income quintiles.

**Figure 2:**
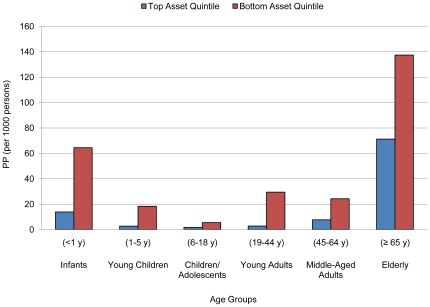
Predicted probabilities (PP) of death by age groups comparing asset quintiles.

**Figure 3:**
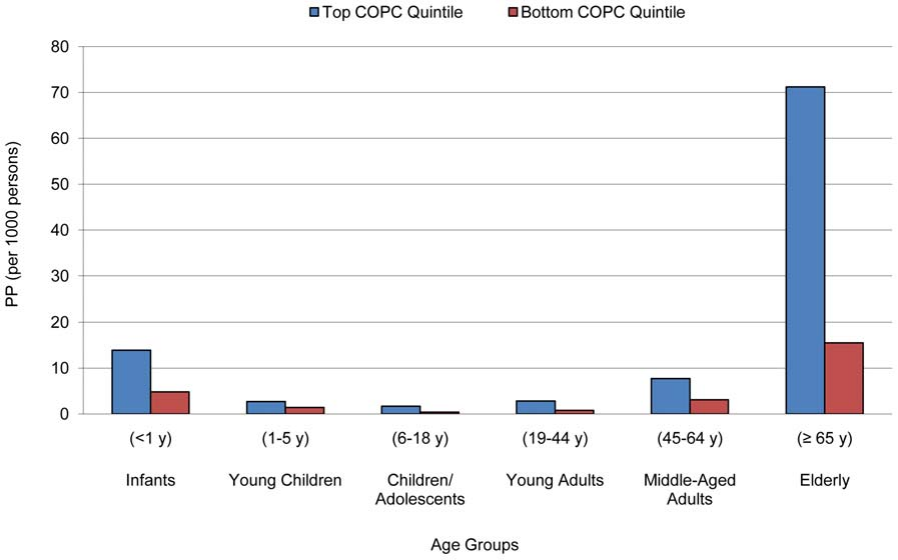
Predicted probabilities (PP) of death by age groups comparing monthly consumption per capita quintiles.

The mortality odds of infants younger than one year were differentially associated with social castes. Furthermore, infants, young children and adolescents up to 18 years old had mortality odds that were differentially associated with quintiles of household assets. Mortality at age 65 and above was significantly associated with being in Scheduled Castes, the bottom income quintile and the bottom assets quintile (Figure 1, Figure 2). No apparent interaction between age and monthly consumption per capita was found (Table 3, Figure 3).

**Table 3:** Predicted Probabilities (95% Confidence Intervals) of Mortality by Socioeconomic Factors Modified by Age, Adjusted for Gender, Urban-Rural Status, Religion, Fixed Effects on States: Indian Human Development Survey, 2004-2005.

	Infants (<1 y)		Young Children (1–5 y)		Children/Adolescents (6–18 y)		Young Adults (19–44 y)		Middle-Aged Adults (45–64 y)		Elderly ( = 65 y)	
	PP^1^	(95% CI)	PP	(95% CI)	PP	(95% CI)	PP	(95% CI)	PP	(95% CI)	PP	(95% CI)
**Caste**												
Brahmin	25.9	(8.7–74.7)**	14.3	(2.7–72.2)**	1.8	(0.5–6.1)	1.8	(0.7–4.7)	11.9	(4.5–31.0)	48.1	(21.9–102.3)
High caste	4.24	(1.1–16.2)	1.8	(0.6–5.3)	1.6	(0.6–4.1)	2.1	(1.0–4.6)	7.7	(3.8–15.5)	35.6	(18.1–68.6)
Other Backward Classes	33.5	(15.4–71.6)**	4.1	(1.8–9.6)	1.2	(0.5–2.6)	2.5	(1.2–5.4)	9.6	(4.7–19.7)	46.4	(23.6–89.0)
Scheduled Castes	30.9	(12.7–73.0)**	4.0	(1.8–8.7)	1.8	(0.8–4.1)	2.3	(1.1–4.9)	8.7	(4.4–17.1)	83.1	(32.1–198.7)*
Scheduled Tribes	38.0	(14.0–99.1)**	7.4	(2.9–18.6)	0.5	(0.1–1.9)**	3.2	(1.4–7.3)	6.9	(2.9–16.1)	46.8	(21.0–100.9)
No caste (Muslim)	22.1	(9.6–50.1)**	4.4	(1.9–10.2)	0.5	(0.1–1.3)**	2.5	(1.0–6.0)	6.1	(2.8–13.4)	50.9	(24.3–103.4)
No caste (Sikh, Jain)	--		--		--		2.0	(0.6–7.0)	6.3	(1.9–20.3)	29.7	(10.7–79.7)
No caste (Christian)	--		1.7	(0.2–13.0)	--		1.8	(0.4–7.0)	12.4	(5.0–30.7)	41.2	(16.8–97.5)
**Income**												
Top quintile	14.1	(5.7–34.3)	2.2	(0.8–5.6)	1.1	(0.5–2.7)	1.6	(0.7–3.5)	5.0	(2.5–10.2)	47.6	(24.4–90.7)
Second quintile	18.0	(7.7–41.6)	2.8	(1.2–12.3)	0.6	(0.2–1.5)	2.1	(1.0–4.5)	5.4	(2.6–11.4)	36.4	(17.9–72.6)
Third quintile	28.4	(12.3–64.3)	6.1	(2.1–17.6)*	1.3	(0.5–3.1)	1.5	(0.7–3.3)	7.6	(3.4–17.0)	60.8	(29.3–122.0)
Fourth quintile	39.4	(16.0–94.2)	5.3	(2.3–12.3)	1.7	(0.6–4.3)	2.7	(1.0–6.8)	14.5	(6.0–34.8)	65.5	(19.5–198.5)
Bottom quintile	37.9	(14.8–93.6)	6.1	(2.6–14.1)	1.4	(0.6–3.5)	4.2	(1.9–9.2)	12.3	(5.5–27.2)	52.2	(24.6–107.1)**
**Household Assets**												
Top quintile	--		--		--		0.6	(0.1–3.9)	6.7	(2.7–16.1)	55.1	(27.0–109.1)
Second quintile	11.5	(3.7–34.7)**	0.5	(0.1–3.3)**	0.2	(0.0–1.7)**	1.7	(0.8–3.5)	7.1	(3.7–13.4)	51.1	(27.8–92.3)
Third quintile	41.6	(19.6–86.4)**	6.0	(2.2–16.2)**	1.3	(0.5–3.1)**	2.1	(1.1–4.2)	14.2	(6.7–29.9)	65.5	(33.5–124.1)
Fourth quintile	49.5	(23.3–101.9)**	8.3	(3.3–20.2)**	2.6	(1.3–5.2)**	5.4	(2.7–10.6)	18.6	(9.0–38.0)	114.4	(49.0–244.9)
Bottom quintile	71.5	(34.1–143.7)**	13.0	(6.3–26.4)**	3.5	(1.6–7.9)**	6.8	(3.3–14.1)	15.9	(7.8–32.3)	93.5	(49.6–169.6)*
**Monthly Consumption per Capita**												
Top quintile	17.4	(7.7–38.9)	2.1	(0.8–5.9)	1.2	(0.5–2.9)	1.7	(0.8–3.5)	6.8	(3.3–13.9)	40.0	(20.5–76.2)
Second quintile	13.4	(5.7–31.2)	1.4	(0.6–3.5)	0.7	(0.3–1.7)	1.4	(0.6–3.2)	4.2	(2.0–8.7)	21.1	(10.7–41.3)
Third quintile	9.3	(3.9–21.9)	1.5	(0.5–4.0)	0.4	(0.2–0.8)	0.5	(0.2–1.0)	2.3	(1.1–4.9)	18.0	(8.8–36.6)
Fourth quintile	9.8	(4.2–22.7)	2.1	(0.9–5.1)	0.2	(0.1–0.5)**	0.9	(0.4–1.9)	2.9	(1.3–6.4)	21.5	(7.3–61.4)
Bottom quintile	5.7	(2.1–15.5)	0.9	(0.4–2.0)	0.3	(0.1–0.8)	0.5	(0.2–1.2)	2.0	(0.9–4.4)	10.0	(4.8–21.0)

1 PP: Predicted probabilities of mortality per 1000 persons given the individual is a male living in the city, who belongs in the High Caste and living in the top quintile of household income, assets ownership and monthly consumption per capita.

*significance with *p*-value <0.10,

**significance with *p*-value <0.05.

### Interaction of Social Castes with Wealth Measures

There were significant interactions found between Scheduled Castes and asset ownership, but not with household income or monthly consumption per capita (Table S3). Upon stratification of social castes, we found significant mortality differentials across asset quintiles in High Caste and in Scheduled Castes (Table 4).

**Table 4:** Odds Ratios of Mortality in Asset Quartiles Stratified by Social Castes, Adjusted for Gender, Urban-Rural Status, Fixed Effects on States: Indian Human Development Survey, 2004-2005.

	High Caste including Brahmin		Other Backward Classes		Scheduled Castes		Scheduled Tribes		No Caste	
	OR	(95% CI)	OR	(95% CI)	OR	(95% CI)	OR	(95% CI)	OR	(95% CI)
**Household Assets**										
Top quartile	1.00		1.00		1.00		1.00		1.00	
Second quartile	1.49	(0.87–2.57)	0.70	(0.38–1.30)	8.54	(1.16–62.68)*	1.35	(0.19–9.55)	0.80	(0.41–1.59)
Third quartile	3.11	(1.27–7.63)*	1.07	(0.59–1.92)	26.22	(3.17–217.25)*	2.80	(0.38–20.91)	1.27	(0.67–2.38)
Bottom quartile	2.42	(1.04–5.60)*	1.32	(0.71–2.43)	29.44	(3.86–224.73)*	2.80	(0.37–21.18)	1.26	(0.64–2.48)

*significance with *p*-value <0.05.

## Discussion

Our analysis has the following findings related to patterns of mortality differential among socioeconomic groups in India. First, the mortality burden associated to lower castes was substantially attenuated after accounting for the individuals' household income and assets. Our analysis showed that infant mortality burden remained associated with social castes. This mirrored previous findings which suggested that there were differential attenuation by economic factors in mortality burdens across life stages [9]. In our case, the importance of economic factors was lesser in infants than older ages. Although lower castes such as Scheduled Castes and Scheduled Tribes are disadvantaged in terms of social standing and materialistic wealth [13], recent studies supported views that economic well-being, such as standard of living, is a more favorable indicator of mortality and morbidity burden than social caste as an intrinsic risk factor [8], [9], [14]. Results from sensitivity analysis provided support that asset ownership, among the three wealth measures, was the most important underlying factor in the mortality differentials observed across social castes (Table S2). Furthermore, individuals belonging to the High Caste and the bottom asset quartile suffered from a higher mortality gap than Other Backward Classes. This suggested that magnitude of inequality across asset ownership may be an additional risk factor (Table 4).

In our study, we utilized three measure of economic well-being to unravel the associations of wealth and mortality patterns. Low household income and asset ownership continued to be strongly associated with increased risk of overall mortality, but asset ownership alone were strongly associated with age-specific mortality. Total household income reflected short term, self-reported wealth of a household. The benefit of higher income may not trickle down to all members of the household at different ages. The data did not reflect significant income effect on mortality risk across age groups (Table 3).

Household asset ownership is a relatively accurate long-term reflection of a household's economic well-being than income. Asset ownership measure has lower recall bias; consumer goods and housing quality can easily be verified by survey administrators. Comparing the two different household wealth indicators, we observed differential mortality gaps across age groups, mainly concentrated in infants and elderly (Figure 1, Figure 2). Furthermore, the mortality gradient of the population over 64 years old was much greater when deprived of asset ownership than of high household income. This suggested that wealth in terms of monetary resources translated less readily to standard of living in elderly than ownership of household goods and housing quality.

Among different household goods and housing qualities, there exists a differential mortality risk reduction for different age groups as well. For instance, an electric fan may reduce risk of malarial infection in infants and young children who stay at home, but not for older children and adults. We observed significant interactions between asset quintiles and age groups 0–18 years but not with older adults, suggesting ownership of particular assets could affect age groups selectively (Table 3).

Consumption captures monthly household expenditures such as staple food cost and seasonal or sporadic expenditures such as contributions to annual festivals, weddings and major medical expenses. Surprisingly, our findings showed significantly lower odds of mortality in individuals from lower quintiles of monthly consumption per capita. Higher quintiles of monthly consumption per capita captured poor households that might need to exert greater marginal effort and percentage of household wealth than rich households to obtain similar daily resources [15]. For example, a woman in an urban center could switch on a tap for potable water in the matter of seconds and a woman living in a rural village may require several hours each morning and afternoon to line up, pump and carry water for daily use, in turn reducing her available income-generating time. Correlations of consumption with income quintiles (r = 0.32) and asset quintiles (r = 0.48) were low, which suggested the subpopulations with low wealth measured by household total income and ownership of assets may not be the similar subpopulations that were consuming the least per capita monthly. On the other hand, high monthly consumption per capita was strongly associated with high mortality risk in elderly individuals (Figure 3). This was likely due to the increased medical expenses at older ages.

The findings of our study need to be considered along with following limitations. Our 2004–2005 update of mortality differential across socioeconomic factors in India was based on cross-sectional survey data. Although it captured only a snap shot of India's mortality patterns, it reinforced previous findings from the NFHS 1998-1999 that economic well-being was a more robust determinant of mortality risk than social caste [8], [16]. However, both findings related to mortality were influenced by recall bias of deaths within the household[17]. Age of the deceased and socioeconomically levels influence under-reporting of deaths differentially. The Sample Registration System (SRS), which is a large-scale demographic survey conducted in India, reports birth rate, death rate and other fertility and mortality indicators at the national and sub-national levels. The crude death rate from IHDS 2004-2005 was 7.4 deaths per 1000 population compared with 8.0 from the 2003 SRS (http://censusindia.gov.in/vital_statistics/Vital_Rates/Vital_rates.aspx; accessed on December 1, 2010). However, infant deaths from IHDS estimate was considerably lower than the SRS estimate. Furthermore, our measures do not capture all dimensions of socioeconomic status. Although multiple studies have illustrated strong link between education levels of the head of household and mother with childhood mortality [5], [16], [18], [19], education levels of individuals who died was not collected from IHDS, thus related aspects such as maternal education level and literacy rate were not included in our analysis. Given the above, conclusions drawn from the mortality analyses presented should qualitatively reflect the underlying patterns of mortality differences across social castes and household economic well-being[5].

### Conclusions

Our study suggests a gradual decrease in importance of social caste as an intrinsic mortality risk. Social caste influences individuals' opportunities to income-generating work and asset ownership. However when adjusted for these economic household measures, the importance of social caste attenuates while the economic measures remain strong indicators of the mortality gap. Overall, mortality differential in India remains salient. Social caste exerts strongest influence in mortality during the first year of life while economically disadvantaged households bear heavier burdens across several age groups.

## Supporting Information

Table S1 Predicted Probabilities of Mortality by Socioeconomic Factors, Adjusted for Gender, Age, Urban-Rural Status, Religion, Fixed Effects on States: Indian Human Development Survey, 2004-2005.
(DOCX)

Table S2 Odds Ratio of Mortality by Social Castes, Adjusted for Income, Assets and Consumption per Capita, Fixed Effects on States: Indian Human Development Survey, 2004-2005.
(DOCX)

Table S3 Statistical Significance (*p -* value) of Mortality in Social Castes modified by Economic Factors, Adjusted for Gender, Urban-Rural Status, Fixed Effects on States: Indian Human Development Survey, 2004-2005.
(DOCX)
