# Supplementary material for: Mortality Burden and Socioeconomic Status in India
Source: PLoS One. 2011 Feb 9;6(2):e16844. doi: 10.1371/journal.pone.0016844 (PMC3036714; doi:10.1371/journal.pone.0016844)
Supplement: Table S1 — Predicted Probabilities of Mortality by Socioeconomic Factors, Adjusted for Gender, Age, Urban-Rural Status, Religion, Fixed Effects on States: Indian Human Development Survey, 2004-2005. (DOCX) [file pone.0016844.s001.docx]

Table S1. Predicted Probabilities of Mortality by Socioeconomic Factors, Adjusted for Gender, Age, Urban-Rural Status, Religion, Fixed Effects on States: Indian Human Development Survey, 2004-2005.

|  | **Unadjusted for SES factors** | | **Adjusted for SES factors** | |
| --- | --- | --- | --- | --- |
|  | **PP^1^** | **(95% CI)** | **PP** | **(95% CI)** |
| **Caste** |  |  |  |  |
| Brahmin | 3.28 | (1.56 - 6.87) | 2.77 | (1.31 - 5.86) |
| High caste | 2.20 | (1.14 - 4.21) | 1.81 | (0.97 - 3.38) |
| Other Backward Class | 3.22 | (1.67 - 6.18) | 2.40 | (1.22 - 4.72) |
| Scheduled Caste | 4.36 | (1.83 - 10.33) | 3.11 | (1.43 - 6.78) |
| Scheduled Tribe | 3.23 | (1.52 - 6.86) | 2.47 | (1.18 - 5.19) |
| No caste (Muslim) | 2.86 | (1.43 - 5.72) | 2.10 | (1.02 - 4.30) |
| No caste (Sikh, Jain) | 1.37 | (0.62 - 3.04) | 1.36 | (0.58 - 3.18) |
| No caste (Christian) | 2.32 | (1.04 - 5.14) | 2.09 | (0.93 - 4.71) |
| **Income** |  |  |  |  |
| Top quintile | 1.52 | (0.61 - 3.82) | 1.81 | (0.97 - 3.38) |
| Second quintile | 1.41 | (0.55 - 3.58) | 1.65 | (0.86 - 3.19) |
| Third quintile | 2.17 | (0.80 - 5.84) | 2.46 | (1.25 - 4.83) |
| Fourth quintile | 2.88 | (0.87 - 9.49) | 3.26 | (1.37 - 7.73) |
| Bottom quintile | 2.67 | (0.98 - 7.26) | 3.00 | (1.48 - 6.05) |
| **Household Assets** |  |  |  |  |
| Top quintile | 2.11 | (1.05 - 4.23) | 1.81 | (0.97 - 3.38) |
| Second quintile | 2.03 | (1.08 - 3.81) | 1.91 | (1.09 - 3.35) |
| Third quintile | 2.92 | (1.55 - 5.52) | 3.14 | (1.69 - 5.84) |
| Fourth quintile | 4.87 | (2.06 - 11.48) | 5.29 | (2.56 - 10.88) |
| Bottom quintile | 5.01 | (2.33 - 10.72) | 5.30 | (2.80 - 10.00) |
| **Monthly Consumption per Capita** |  |  |  |  |
| Top quintile | 4.02 | (2.05 - 7.88) | 1.81 | (0.97 - 3.38) |
| Second quintile | 3.12 | (1.57 - 6.22) | 1.09 | (0.55 - 2.13) |
| Third quintile | 2.51 | (1.18 - 5.31) | 0.71 | (0.36 - 1.41) |
| Fourth quintile | 3.64 | (1.34 - 9.90) | 0.89 | (0.39 - 2.02) |
| Bottom quintile | 2.19 | (0.98 - 4.88) | 0.49 | (0.24 - 1.00) |

^1^PP: Predicted probabilities of mortality if the individual is a 19-44 year old male living in the city who belongs in the High Caste and living in the top quintile of household income, assets ownership and monthly consumption per capita.
