# Supplementary material for: Mortality Burden and Socioeconomic Status in India
Source: PLoS One. 2011 Feb 9;6(2):e16844. doi: 10.1371/journal.pone.0016844 (PMC3036714; doi:10.1371/journal.pone.0016844)
Supplement: Table S2 — Odds Ratio of Mortality by Social Castes, Adjusted for Income, Assets and Consumption per Capita, Fixed Effects on States: Indian Human Development Survey, 2004-2005. (DOCX) [file pone.0016844.s002.docx]

Table S2. Odds Ratio of Mortality by Social Caste, Adjusted for Income, Assets and Consumption per Capita, Fixed Effects on States: Indian Human Development Survey, 2004-2005.

|  | **Unadjusted for SES factors** | | **Adjusted for Income only** | | **Adjusted for Assets only** | | **Adjusted for Consumption per capita only** | | **Adjusted for all wealth factors** | |
| --- | --- | --- | --- | --- | --- | --- | --- | --- | --- | --- |
|  | **OR** | **(95% CI)** | **OR** | **(95% CI)** | **OR** | **(95% CI)** | **OR** | **(95% CI)** | **OR** | **(95% CI)** |
| **Caste** |  |  |  |  |  |  |  |  |  |  |
| Brahmin | 1.50 | (0.89 - 2.53) | 1.52 | (0.91 - 2.56) | 1.54 | (0.92 - 2.59) | 1.48 | (0.87 - 2.50) | 1.54 | (0.92 - 2.57) |
| High caste | 1.00 |  | 1.00 |  | 1.00 |  | 1.00 |  | 1.00 |  |
| Other Backward Classes | 1.47 | (1.09 - 1.98)* | 1.33 | (0.97 - 1.81) | 1.28 | (0.94 - 1.75) | 1.59 | (1.20 - 2.11)* | 1.33 | (0.99 - 1.79) |
| Scheduled Castes | 1.99 | (1.36 - 2.92)* | 1.70 | (1.19 - 2.45)* | 1.63 | (1.12 - 2.35)* | 2.25 | (1.59 - 3.17)* | 1.72 | (1.23 - 2.41)* |
| Scheduled Tribes | 1.47 | (1.02 - 2.13)* | 1.23 | (0.85 - 1.79) | 1.17 | (0.80 - 1.71) | 1.80 | (1.26 - 2.58)* | 1.37 | (0.95 - 1.98) |
| No caste (Muslim) | 1.30 | (0.91 - 1.87) | 1.18 | (0.81 - 1.71) | 1.10 | (0.75 - 1.61) | 1.43 | (1.02 - 2.02)* | 1.16 | (0.81 - 1.66) |
| No caste (Sikh, Jain) | 0.63 | (0.36 - 1.09) | 0.70 | (0.40 - 1.22) | 0.79 | (0.45 - 1.36) | 0.57 | (0.32 - 1.01) | 0.75 | (0.43 - 1.31) |
| No caste (Christian) | 1.06 | (0.61 - 1.82) | 1.06 | (0.62 - 1.80) | 1.16 | (0.66 - 2.02) | 1.06 | (0.63 - 1.76) | 1.16 | (0.70 - 1.91) |

*significance with *p*-value < 0.05
