# Supplementary material for: Mortality Burden and Socioeconomic Status in India
Source: PLoS One. 2011 Feb 9;6(2):e16844. doi: 10.1371/journal.pone.0016844 (PMC3036714; doi:10.1371/journal.pone.0016844)
Supplement: Table S3 — Statistical Significance (p - value) of Mortality in Social Castes modified by Economic Factors, Adjusted for Gender, Urban-Rural Status, Fixed Effects on States: Indian Human Development Survey, 2004-2005. (DOCX) [file pone.0016844.s003.docx]

Table S3. Statistical Significance (*p -* value) of Mortality in Social Castes modified by Economic Factors, Adjusted for Gender, Urban-Rural Status, Fixed Effects on States: Indian Human Development Survey, 2004-2005.

|  | **Brahmin** | **Other Backward Classes** | **Scheduled Castes** | **Scheduled Tribes** | **No caste (Muslim)** | **No caste**  **(Sikh, Jain)** | **No caste (Christian)** |
| --- | --- | --- | --- | --- | --- | --- | --- |
| **Income** |  |  |  |  |  |  |  |
| Top quintile |  |  |  |  |  |  |  |
| Second quintile | NS | NS | NS | NS | NS | NS | NS |
| Third quintile | NS | NS | NS | NS | NS | NS | NS |
| Fourth quintile | <0.01 | NS | NS | NS | 0.02 | NS | NS |
| Bottom quintile | NS | NS | NS | NS | NS | < .0001 | NS |
| **Household Assets** |  |  |  |  |  |  |  |
| Top quartile |  |  |  |  |  |  |  |
| Second quartile | NS | NS | 0.07 | NS | NS | NS | NS |
| Third quartile | NS | NS | 0.03 | NS | NS | NS | NS |
| Bottom quartile | NS | NS | 0.01 | NS | NS | <.0001 | NS |
| **Monthly Consumption per Capita** |  |  |  |  |  |  |  |
| Top quintile |  |  |  |  |  |  |  |
| Second quintile | NS | NS | NS | NS | NS | NS | NS |
| Third quintile | NS | 0.03 | 0.10 | NS | NS | NS | NS |
| Fourth quintile | 0.01 | NS | NS | NS | NS | < .0001 | NS |
| Bottom quintile | NS | NS | NS | NS | NS | < .0001 | NS |

Reference group: High caste

NS: not significant with *p*-value > 0.10
